# Reply: Comment on BIA-ALCL: Comparing the Risk Profiles of Smooth and Textured Breast Implants

**DOI:** 10.1007/s00266-023-03426-8

**Published:** 2023-06-12

**Authors:** Eric Swanson

**Affiliations:** Swanson Center, 11413 Ash St, Leawood, KS 66211 USA

## Abstract

*Level of Evidence V* This journal requires that authors assign a level of evidence to each article. For a full description of these Evidence-Based Medicine ratings, please refer to the Table of Contents or the online Instructions to Authors www.springer.com/00266.

Drs. Longo, Giacolone, and Cervelli [1] rebut several of the points made in a comparison of the risk profiles of smooth and textured breast implants published by Swanson [2]. In their referenced article [3], the authors commented, “there is no definite evidence that preclude (*sic*) any association between smooth implants and the pathogenesis of Breast Implant-Associated Anaplastic Large-Cell Lymphoma (BIA-ALCL ).” The authors point to the fact that among 1130 medical device reports submitted to the US Food and Drug Administration (FDA) of patients diagnosed with BIA-ALCL, 295 (26%) do not specify the implant surface [4]. Notably, the FDA has no documentation of a case occurring in a woman known to have a history of only smooth devices [4–6]. Despite thousands of plastic surgeons around the globe on the lookout for a case of BIA-ALCL in a woman implanted only with smooth implants, there is still no published case report. For practical purposes, this information is all surgeons need to know to eliminate the risk of BIA-ALCL in their patients who choose breast implants for cosmetic augmentation or reconstruction [7]. Going forward, no texture means no BIA-ALCL. The conclusion regarding the categorical (not relative) difference in risk is inescapable [2].

To be fair, there is a possibility that a case of BIA-ALCL will 1 day be diagnosed in a woman with a smooth implant-only history. Such a case will no doubt bring relief to surgeons “hunting” for the first BIA-ALCL smooth case [5]. After all, this T-cell lymphoma occurs sporadically in patients without implants, albeit with a frequency on the order of 1 in 4 million [8]. The coincidental occurrence of such a tumor in the breast of a woman with a smooth implant and no previous textured devices is theoretically possible. It is also possible that such a case will be reported simply because errors are possible in recording implant characteristics.

The authors correctly comment that the pathogenesis of BIA-ALCL remains unknown, like many cancers. However, the epidemiological evidence of a link to texturing is conclusive, to the extent that in 2021 the Scientific Committee on Health, Environmental and Emerging Risks (SCHEER) group concluded that there was moderate (which is deemed to be good) evidence of a causal relationship between textured devices and BIA-ALCL [9].

Longo et al. [1] believe that to conclude that the risk of BIA-ALCL is zero when using smooth implants, two assumptions are needed: (1) patients with BIA-ALCL and an unknown history regarding implant type all had textured devices at some point, and (2) in patients with a history of both smooth and textured devices, the risk is attributable to the textured devices and not the smooth ones, and “the disease is certainly caused by a textured device implanted at the onset of symptoms.”

Of course, speculation about the type of implants in women with an unknown or incomplete implant history is not helpful. One cannot assume that one or more of these women had a smooth-only implant history or that none of them did. Only cases with reliable data (i.e., full implant history and identification of surface types) can be trusted. With regard to the second point, a median 8-year lag time between implant insertion and a BIA-ALCL diagnosis is well known [4]. Understandably, the implant type may change during this time interval; women frequently have implants replaced [10]. The pertinent issue is simply whether the patient was ever exposed to textured devices. The question is not whether a patient has ever been exposed to smooth devices because, among millions of women implanted to date worldwide with smooth devices only, not one is known to have developed BIA-ALCL [4–6, 10].

Ironically, Longo et al. [1] recommend against any “misleading interpretation” of the evidence. The evidence linking textured implants to BIA-ALCL remains unchanged since the landmark 2015 publication by Brody et al. [11]. Eight years have elapsed and the evidence for a link to this surface type has grown only more solid. A name change is justified. It is time to amend the name to Textured Implant-Associated Anaplastic Large-Cell Lymphoma (TIA-ALCL) [12]. A name change is not merely an academic matter or one of semantics. Failure to recognize a categorical difference in risk has harmful consequences for women [2, 12]. The authors’ guidelines [3] are a testament to the problem created when labeling this disease “Breast Implant-Associated,” bundling together all types of implants and surfaces, and casting too wide a net.

New boxed warnings (i.e., the warnings are outlined in a bold rectangle for emphasis) frighten women considering breast implants for either cosmetic augmentation or reconstruction. A recent survey showed that the effect is to double the number of women who believe breast implants are unsafe, from 29 to 58%, and reduce by 50% the number of women likely to consider having breast implants [13]. After reading the warning, women are more willing to consider autologous alternatives.

Our duty is to inform patients of foreseeable risks, not every conceivable risk, of surgery [12]. Despite their problems, breast implants are known to effectively boost women’s self-esteem and quality of life. The number of women who are self-conscious about their breasts drops dramatically [14]. To unnecessarily scare women considering breast augmentation using smooth implants does them a disservice [2]. In the USA, textured devices have been largely abandoned [2, 6]; the boxed warning is a moot point if textured devices are not implanted. An intervention (the boxed warning) intended to help and protect women serves instead to alarm them of a risk that has never even been reported and deny them an opportunity for increased happiness and improved self-esteem.

Longo et al. [1, 3] mandate autologous reconstruction in all women treated for BIA-ALCL. This recommendation differs from existing recommendations that replacement implants may be used, but only smooth, not textured, devices [15]. This prohibition on implants is meant to avoid a *spectacularly* unlikely, in fact still hypothetical, case of BIA-ALCL occurring in a woman with this diagnosis after replacement with a smooth implant. The authors defend this position by opining that patients who have already developed BIA-ALCL may be different, and perhaps more genetically disposed to this disease, which is certainly possible. However, the evidence shows that a genetic susceptibility alone is not sufficient to produce the disease in women treated with smooth implants [4–6, 10]. A textured device is needed to trigger it.

Patients who have had a cosmetic breast augmentation, developed BIA-ALCL, and who undergo bilateral explantation, are not given an implant option by Longo et al. [1, 3]. They are directed to undergo either fat injection, a free flap reconstruction, or a fat-augmented latissimus dorsi (FALD) flap instead. Women who developed BIA-ALCL after breast augmentation are converted from cosmetic breast patients to reconstructive patients—introducing extra cost, morbidity, scarring, and generally less favorable results.

A recommendation for autologous alternatives does not consider the whole new set of complications introduced by non-implant reconstruction. Free flap reconstruction adds a small but non-hypothetical risk of mortality from anastomotic hemorrhage, venous thromboembolism, and anesthesia risk from this major procedure [16]. By contrast, the risk of implant surgery is negligible. A recent retrospective series of almost 100,000 breast implant procedures included no fatalities [17]. Many women are too lean for successful fat injection. Attempts to maximize the fat harvest are likely to create donor site deformities. Latissimus dorsi flaps may not provide adequate volume, especially in patients with larger breasts, even when augmented by fat [18].

The authors’ treatment algorithm [3] keeps an important option off the table for patients. Women who wish to avoid the limitations of autologous reconstruction deserve to be given this option. Surgeons should be wary about making recommendations that greatly intensify the nature of the intervention, risk, and the financial implications for patients.

Longo et al. [1] insist the evidence is still insufficient to “rule out smooth implants as causative agents.” The authors believe future evidence may still implicate smooth implants as a causative agent for BIA-ALCL, while disputing the sufficiency of existing evidence implicating textured surfaces as a causative agent, as opposed to a correlation. This surprising opinion is at odds with the conclusions of the SCHEER group [9] and the actions of the FDA and virtually all health regulatory agencies across the globe [7]. Even Allergan (an AbbVie Company, North Chicago, Ill.) did not dispute the FDA request for withdrawal of macrotextured implants from the marketplace [7]. The authors believe that those who disagree with them are given to personal thoughts or convictions, not to the most reliable scientific evidence [1].

Oddly, the authors [1] mention that their precautionary principle of denying implants to women who have been diagnosed with BIA-ALCL is “detailed in Article 191 of the Treaty on the Functioning of the European Union.” The referenced article does not pertain to medical care. It provides that preventive action be taken so “that environmental damage should as a priority be rectified at source and that the polluter should pay” [19].

Interestingly, the science is developing. As recently as 2017, infection was considered a leading theory for the cause of BIA-ALCL [20]. Today, chronic inflammation, surface texturing, particulates, and a genetic predisposition are believed to be involved in the pathogenesis [6, 8]. In a widely cited 2021 animal study, Doloff et al. [21] showed that surface topography mediates the immune response to breast implants. Textured implants provoke inflammation and a foreign body response. A macrotextured implant creates an immunocompromised zone around the device [6, 21].

Conflict of interest is relevant [22]. According to the journal website, a co-author of the original article (G. Curigliano) is also the co-editor of the journal in which the article was published. There is no mention of recusal of this editor during the peer review process. If the lead author (B. Longo) is married to a plastic surgeon (A. Campanale) who is a medical director at the Italian Ministry of Health in the Department of Drugs and Medical Devices, this familial relationship should be disclosed.

In their letter, Longo et al. [1] comment that their recommendations were drafted in collaboration with the Italian Ministry of Health. In their article [3], the authors report that their consensus statement was developed in accordance (as opposed to collaboration) with the latest directives of the Italian Ministry of Health. Do the authors suggest that the Ministry of Health endorses their viewpoint regarding lack of a causal relationship between textured devices and BIA-ALCL? If so, such a perspective stands in stark contrast to the conclusions of the SCHEER report [9].

Sackett [23], a founder of modern evidence-based medicine, cautioned, “The first sin committed by experts consists in adding their prestige and their position to their opinions, which give the latter far greater persuasive power than they deserve on scientific grounds alone.” The opinion of a panel of experts ranks lowest among the levels of evidence. Evidence-based medicine does not acknowledge scientific validity based on the endorsement of a government authority. It recognizes only the facts [24].

Our scientific journals represent the proper forum for debate on important issues such as this one that has a profound effect on women’s quality of life. Differing viewpoints should be welcome [25]. Plastic surgeons can judge the merits and make recommendations that reflect the best available evidence, and the best interests of their patients.
